# Prevalence and Symptomatic Benefit of Antibiotic Use in End-of-Life Patients in Saudi Arabia: An Observational Cohort Study

**DOI:** 10.1177/08258597251336821

**Published:** 2025-05-08

**Authors:** Seham Radhi, Mohammed A. Alamri, Abdurrahman A. Ksibati, Nadia A. Shahda, Abdullaha I. Alsuhail, Mustafa Zakkar, Ibrahim Antoun

**Affiliations:** 1 Department of Palliative Care, King Fahed Medical City, Riyad, Saudi Arabia; 2 College of Medicine, 101686 Alfaisal University, Riyad, Saudi Arabia; 3 Department of Cardiovascular Sciences, 4488 University of Leicester, Leicester, UK

**Keywords:** infections, antibiotics, palliative care, cancer, end-of-life care, symptom control

## Abstract

**Objectives:** Antibiotic use in patients with advanced cancer at the end of life (EoL) is common but controversial, with limited evidence on its efficacy in improving symptoms. This study aimed to evaluate the prevalence of antibiotic use during the final 30 days of life in patients with advanced cancer and its impact on symptom improvement within a palliative care setting in Saudi Arabia. **Methods:** A retrospective cohort study was conducted in the palliative care department of King Fahad Medical City, Riyadh, Saudi Arabia. The study included all advanced cancer patients who received inpatient palliative care and died between January 2022 and March 2023. Medical records data were analyzed to assess infection-related symptoms, antibiotic use, and symptom improvement 3 days (D3) post-diagnosis. **Results:** A total of 220 patients were included, with a mean age of 61 ± 17 years and a mean palliative performance scale of 37%. Antibiotics were prescribed to 89% of patients, primarily empirically (82%). Piperacillin/tazobactam (53%) and meropenem (17%) were the most commonly used antibiotics. Symptom improvement at D3 was observed in 54% of symptomatic patients (n = 95). Improvements were significant for fever (42% to 15%, *P* < .001), pain (58% to 37%, *P* < .001), cough (16% to 7%, *P* = .004), and shortness of breath (32% to 20%, *P* = .003). The logistic regression model analysis identified no significant predictors of symptom improvement. **Conclusion:** Antibiotics are widely used EoL care for advanced cancer patients, but their impact on symptom improvement is modest. The findings underscore the need for judicious antibiotic use, guided by individualized care goals and interdisciplinary collaboration, to optimize symptom management while minimizing unnecessary interventions.

## Introduction

Antibiotic use in patients with advanced cancer approaching the end of life (EoL) is a complex and debated aspect of palliative care. While antibiotics are commonly prescribed in this population, their role in improving quality of life versus their potential to prolong suffering or lead to adverse effects remains uncertain. The ethical and clinical dilemmas surrounding antibiotic use are compounded by the difficulty in diagnosing infections in this setting, as symptoms such as fever, cough, or delirium may arise from cancer progression, treatment side effects, or noninfectious causes.

Existing literature highlights the widespread use of antibiotics in palliative care, with a large variation of antibiotics used during the last days of life.^1^ Studies from diverse settings, including hospice and inpatient care, have shown considerable variation in prescribing practices, often influenced by cultural, institutional, patient preferences, and individual clinician factors.^2^

Concerns about antimicrobial resistance, particularly in healthcare settings with high antibiotic consumption, further complicate the balance between potential benefits and harms. A growing body of evidence questions the efficacy of antibiotics in palliative care, with some studies suggesting minimal symptomatic relief and a lack of significant improvement in survival.^3^ Furthermore, infectious disease specialists are often limited in their involvement in EoL care decisions, leaving much of the responsibility to palliative care and oncology teams.

While global research has established these concerns, the specific context of antibiotic use in palliative care within the Middle East remains understudied. The Middle Eastern healthcare system faces unique challenges, including high rates of antimicrobial resistance, cultural expectations regarding aggressive treatment at EoL, and differing healthcare policies compared to Western nations.^4^ Understanding antibiotic prescribing practices in Saudi Arabia is essential to tailoring appropriate guidelines that balance symptom relief with responsible antimicrobial stewardship.

This study seeks to fill this gap by providing empirical data from a Saudi Arabian tertiary cancer center, offering insights that can guide more informed and culturally sensitive prescribing practices. The 3 objectives of this retrospective study are to determine the prevalence of antibiotic use among EoL cancer patients in a palliative care setting, assess the impact of antibiotic treatment on symptom relief, and evaluate potential predictors of symptom improvement following antibiotic therapy.

## Methods

This retrospective study was conducted within the palliative care department of a 91-bed Comprehensive Cancer Center at King Fahad Medical City (KFMC) in Riyadh, Saudi Arabia. The study cohort consisted of all patients with advanced cancer who received palliative care and died within the inpatient setting between January 2022 and March 2023. Data were obtained from the patient's medical records spanning the 30 days preceding death. Infective symptoms were defined as the presence of at least one of the following: fever (>37.8°C, measured in the ear), cough, shortness of breath, delirium, or pain. Diagnosis of infection was based on clinician assessment, supported by laboratory markers (such as leukocytosis or elevated inflammatory markers), microbiological cultures, and radiological findings where available. The Epic system and the palliative care program database served as data sources. All data were recorded using a standardized, predesigned data collection form. The study was written according to STROBE guidelines.^5^ The research reported in this article adhered to the Declaration of Helsinki. The study protocol was reviewed and approved by the Institutional Review Board of KFMC (IRB No. 24-119). Informed consent was waived due to the study's retrospective nature, and the use of anonymized data obtained from the Epic electronic health record system.

### Outcomes

The primary outcome of this study was the prevalence of antibiotic use during the final 30 days of life in patients with advanced cancer receiving inpatient palliative care. Prevalence was defined as the proportion of patients who received at least one antibiotic prescription during this period, regardless of the indication. The secondary outcome of this study was to evaluate predictors of symptom improvement at day 3 (D3) following antibiotic initiation. Symptom improvement was defined as a documented reduction in the severity of at least one infection-related symptom (fever, cough, shortness of breath, delirium, or pain) based on clinician assessment in medical records. A fever reduction was defined as a temperature decrease to below 37.8°C without using antipyretics. Improvement in respiratory symptoms (cough or shortness of breath) was determined by clinician-reported relief and decreased need for supplemental oxygen or nebulization. Delirium improvement was assessed based on documented clinical stabilization, agitation reduction, or orientation status improvement. Pain improvement was defined as a reduction in opioid or adjunctive analgesic dosing requirements or a decrease in documented pain scores.

### The Rationale for Sample Size Requirements

The primary objective was to estimate the prevalence of antibiotic use (defined as yes/no) within the study population. A sample size calculation, targeting a 95% confidence level and a margin of error not exceeding 0.15 in either direction, yielded a required sample size of 220 patients ***.***

### Statistical Analysis

Continuous variables are represented as mean and standard deviation, whereas categorical variables are expressed as counts and percentages (%). Pearson χ² or Fisher exact test was utilized to compare categorical variables between groups, while the Student *t* test was applied to compare continuous variables. Univariable and multivariable logistic regressions were employed to examine the relationship between antibiotic use and symptom improvement. We hypothesized that specific demographics, comorbidities, and characteristics would influence symptom improvement. Consequently, a base model was developed to evaluate the incremental value of comorbidities and antibiotics use significantly associated with symptom improvement. Statistically significant variables identified in the univariate analysis were incorporated into the multivariable analysis. A 2-sided *P*-value of <.05 was deemed statistically significant. Statistical analysis was conducted using GraphPad Prism V10.3 for Mac.

## Results

### Patient Demographics and Infection Details

The study included 220 patients with advanced cancer. Table 1 presents the demographic and clinical characteristics of the cohort. The mean age was 61 ± 17, and 102 (46%) were males. The mean palliative performance scale was 37%. The most common malignancy was gastrointestinal (17%), followed by breast (14%). Symptoms were present in 184 patients (84%) before antibiotics initiation. Venous blood culture was positive in 94 patients (53%), and 98 patients (45%) had leukocytosis. Of the 220 patients included in the study, 196 (89%) received antibiotics during the final 30 days of life, with 161 (82%) of these prescriptions being empirical. This high prevalence underscores the entrenched role of antibiotics in palliative care despite ongoing debates about their overall benefit in symptom management. The frequent use of empirical antibiotics suggests a reliance on broad-spectrum coverage, often initiated without microbiological confirmation, which may reflect clinical uncertainty in diagnosing infections in this patient population. The most used antibiotic was piperacillin/tazobactam in 104 patients (53%), followed by meropenem in 34 patients (17%). Prescribing was primarily conducted by palliative care teams (44%) and medical oncology teams (35%), with infectious disease specialists involved in 11% of shared decision-making instances. Unlocated infections were most prevalent in 85 patients (43%), and the most commonly located infection was in the respiratory tract in 48 patients (24%), followed by the urinary tract in 39 patients (20%). Data regarding imaging were available in 145 patients, of which 67 (86%) had radiological evidence of infection. This distribution suggests that respiratory and urinary infections remain a major clinical concern in palliative care, aligning with previous studies highlighting the challenge of distinguishing true infection from cancer-related inflammation or treatment side effects.

**Table 1.** Baseline Characteristics for the Study Cohort.

Demographics, n (%) or mean ± SD	
Age (years)	61 ± 17
Palliative Performance Scale (%)	37 ± 13
Males	102 (46%)
Malignancy location, n (%)	
Urological malignancy	13 (6%)
Unknown primary	2 (1%)
Thyroid	4 (2%)
Sarcoma	6 (3%)
Pancreas	19 (9%)
Lung	15 (7%)
Liver	20 (9%)
Kidney	4 (2%)
Hematological	10 (5%)
Head and neck	14 (6%)
Gynecological	16 (7%)
Gastrointerstinal tract	38 (17%)
Central nervous system	15 (7%)
Breast	31 (14%)
Biliary system	12 (5%)
Adrenal	0.50%
Site of infection (n = 196), n (%)	
Wound	6 (3%)
Urinary tract	39 (20%)
Respiratory tract	48 (24%)
Abdominal	18 (9%)
Unlocated	85 (43%)
Infection details and risk factors, n (%)	
Symptoms prior to antibiotics^a^	184 (84%)
Leucocytosis	98 (45%)
Presence of a foreign body^b^	155 (70%)
Positive culture^c^	94 (53%)
Symptoms after 3 days of antibiotics^d^	65 (52%)
Antibiotics used (n = 196), n (%)	
Empirical use	161 (82%)
Cefepime	5 (3%)
Ceftriaxone	28 (14%)
Ciprofloxacin	4 (2%)
Meropenem	34 (17%)
Pipracillin/Tazobactam	105 (53%)
Vancomycin	6 (3%)
Others	14 (7%)

Abbreviation: SD, standard deviation.

a Symptoms included fever, delirium, cough, new pain, and shortness of breath.

b Foreign bodies included: ascitic drain, external catheters or feeding tubes, ventriculoperitoneal shunts, or common bile duct stent.

c Culture was taken from 176 patients.

d Calculated out of total of patients who had symptoms prior to antibiotics, n = 196.

### Outcomes

**Figure 1.**
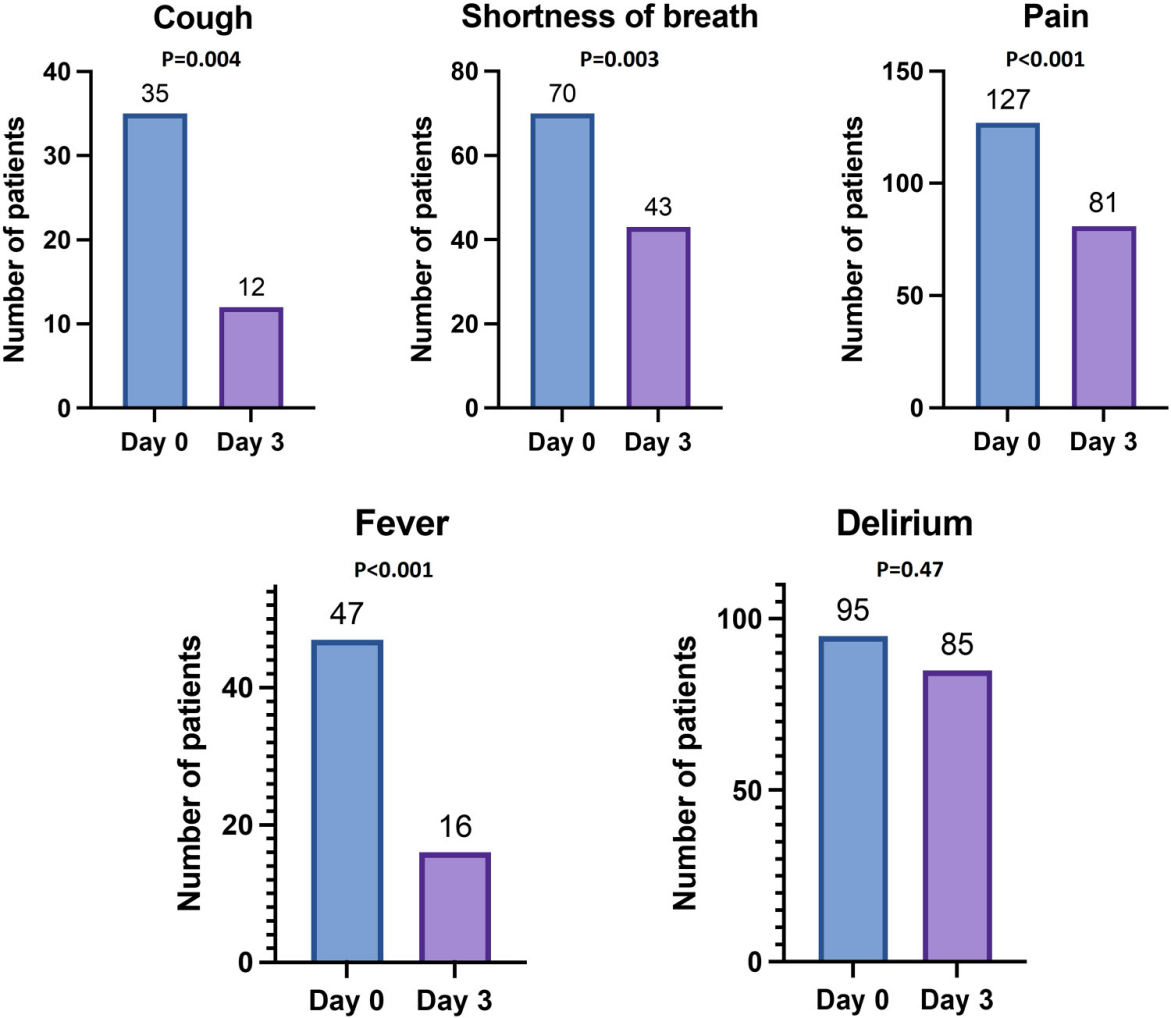
Comparison between symptoms at day 0 and day 3 after starting antibiotics.

Of the 184 symptomatic patients, 91% were on antibiotics. Symptomatic improvement at D3 happened in 95 patients (54%). Details of symptom improvement are demonstrated in Figure 1. The improvement occurred in pain (58% to 37%, *P* < .001), cough (16% to 7%, *P* = .004), shortness of breath (32% to 20%, *P* = .003), and fever (42% to 15%, *P* < .001). The univariable logistic regression model shown in Table 2 did not demonstrate a predictive variable for symptom improvement at D3.

**Table 2.** Logistic Regression Regarding Predictors for Symptoms Improvement in the Study Cohort.

	Univariable regression	
Variable	OR (95% CI)	*P* value
Age (for every 10 years increase)	1 (0.9-1)	.91
Females (vs males)	1.1 (0.8-1.7)	.75
Antibiotics use (yes vs no)	1 (0.9-1)	.96
Leucocytosis (yes vs no)	0.8 (0.4-1.4)	.43
PPS (for every 10% increase)	1 (0.97-1)	.68
Foreign material (yes vs no)	1.1 (0.6-2)	.86
Positive culture (yes vs no)	1.5 (0.8-2.9)	.2
Empirical antibiotics (vs targeted antibiotics)	0.6 (0.3-1.3)	.13
Wound infection (yes vs no)	1.1 (0.8-1.9)	.6
Urinary tract infection (yes vs no)	0.9 (0.5-1.6)	.7
Respiratory tract infection (yes vs no)	1.3 (0.8-2.1)	.4
Intraabdominal infection (yes vs no)	1 (0.6-1.8)	.8
Unlocated infection (yes vs no)	1.2 (0.7-2.1)	.3
Infection documented by imaging (yes vs no)	1.3 (0.5-2.2)	.24

Abbreviations: CI, confidence interval; OR, odds ratio; PPS, Palliative Performance Scale.

## Discussion

This is the first study to address the frequency and symptomatic benefit of antibiotic use in palliative care settings in the Middle East. The study's main finding is that antibiotic use was frequent in palliative care settings with a presumed infection. However, antibiotics were not independently responsible for improving symptoms at D3. The primary outcome of this study, namely the prevalence of antibiotic use in EoL care, is crucial in understanding contemporary palliative care practices. The high rate of antibiotic use (89%) indicates the common approach to managing infections in terminally ill cancer patients, despite ongoing debates surrounding its benefits. Evaluating antibiotic prevalence in this context facilitates a deeper understanding of prescribing behaviors, the role of empirical treatment, and the impact of antibiotics on patient comfort during their final days. Considering that symptom relief, rather than curative treatment, is the principal goal of palliative care, analyzing antibiotic prescribing patterns can lead to more tailored, patient-centered approaches while minimizing unnecessary medical interventions.

The use of antibiotics in patients with advanced cancer nearing the EoL presents a multifaceted dilemma in palliative care, characterized by ethical considerations, clinical challenges, and varying practices across different healthcare settings. The literature indicates a significant prevalence of antibiotic use in this population, often without clear evidence of benefit, which raises questions about the appropriateness of such interventions. For instance, similar to our results, a previous study found that nearly 90% of hospitalized patients with advanced cancer received antibiotics during the last week of life despite limited data supporting their effectiveness in alleviating infection-related symptoms.^6^ This trend is echoed in various studies highlighting the inconsistency in prescribing practices influenced by cultural, institutional, and individual clinician factors.^7,8^

One important aspect to consider when prescribing antibiotics is the nature of infections in these patients. The most common infections in EoL cancer patients include respiratory infections, urinary tract infections, and bloodstream infections, often associated with underlying immunosuppression and prolonged hospitalization. The etiology of infections in this population is typically bacterial, with Gram-negative organisms such as *Escherichia coli and* Enterococcus and Gram-positive bacteria such as *Staphylococcus aureus* and *Enterococcus* species.^9,10^ Fungal infections, particularly candidemia, can also occur in patients with prolonged hospital stays or recent chemotherapy.^9,10^

The empirical choice of antibiotics in palliative care settings depends on multiple factors, including suspected site of infection, local antibiogram data, patient-specific risk factors for multidrug-resistant organisms, and institutional policies. Our study used broad-spectrum antibiotics such as piperacillin/tazobactam and meropenem, reflecting the standard practice of covering Gram-positive and Gram-negative pathogens in high-risk patients. However, concerns about antimicrobial resistance highlight the need for careful antibiotic selection, balancing the potential symptomatic benefit against the risks of resistance and adverse effects.^4^

Decisions regarding when to initiate antibiotic therapy in palliative care patients are often complex and should be guided by clear clinical criteria. Common indications include evidence of a clinically significant infection with associated distressing symptoms, laboratory or radiological confirmation, and an expected symptomatic benefit from treatment. In contrast, antibiotics may not be warranted in cases where infection is not causing significant discomfort, prognosis is extremely limited, or therapy is unlikely to alter the clinical trajectory.^11^ Our study's lack of predictive variables for symptomatic improvement further underscores the need for individualized decision-making focusing on patient comfort and overall care goals.

The ethical considerations of antibiotic use in palliative care extend beyond their potential clinical benefits to encompass patient autonomy, informed decision-making, and alignment with EoL care goals.^12,13^ Current clinical practice guidelines in palliative care emphasize patient-centered decision-making, where antibiotics should be based on clear therapeutic goals rather than routine prescribing habits. While symptom relief remains a primary justification for antibiotic use, the limited evidence supporting their effectiveness necessitates more robust discussions between healthcare providers, patients, and families.

Patient autonomy is a key ethical principle in palliative care, yet antibiotic prescribing often occurs without fully engaging patients or their surrogates in discussions about expected benefits and potential drawbacks. Given the high rates of antibiotic use observed in this study, structured decision-making frameworks that incorporate the patient's prognosis, personal preferences, and symptom burden are needed to determine whether antibiotics should be initiated or withheld.

Furthermore, the broader implications of widespread antibiotic prescribing include increased antimicrobial resistance and potential adverse effects that may worsen rather than alleviate patient suffering. Antibiotic stewardship should not be overlooked in a setting where palliative care aims to maximize comfort. Integrating infectious disease specialists into palliative care teams may help refine prescribing practices and ensure that antibiotic use aligns with patient-centered and public health priorities.

The findings from this Saudi Arabian study contribute to this discourse by providing empirical data on antibiotic prescribing patterns in a palliative care setting. Despite high rates of antibiotic use, only 54% of symptomatic patients demonstrated improvement in their symptoms by day 3 of treatment, suggesting that the benefits of antibiotics may not be as pronounced as often assumed.^14^ This aligns with previous research indicating that antibiotic treatment may not significantly enhance symptom relief or survival in terminally ill patients.^15,16^ Moreover, the involvement of infectious disease specialists in EoL care decisions appears to be limited, which may contribute to the high rates of antibiotic prescriptions without adequate justification.^15,17^ The reliance on palliative care and oncology teams for prescribing decisions highlights the need for interdisciplinary collaboration to ensure that antibiotic use aligns with best practices and patient-centered care principles.^7,8,18^ As the study indicates, understanding the patterns and drivers of antibiotic use in palliative care is crucial for developing guidelines that prioritize patient comfort and minimize unnecessary interventions. As the study indicates, understanding the patterns and drivers of antibiotic use in palliative care is crucial for developing guidelines that prioritize patient comfort and minimize unnecessary interventions.

It is noted that there are limited predictive variables for symptom improvement, but several confounding factors could have influenced the findings. Variability in individual responses to treatment, including differences in baseline functional status, immune response, and underlying comorbidities, may have played a role in symptom resolution. Additionally, the concurrent use of supportive care measures such as analgesics, antipyretics, and oxygen therapy could have contributed to symptom improvement, making it difficult to isolate the specific effect of antibiotics. Clinician decision-making, informed by subjective assessments and institutional practices, may have also influenced the variability in reported outcomes. Accounting for these factors in future prospective studies may provide a clearer understanding of the true impact of antibiotics in palliative care settings. This underscores the need for individualized decision-making, guided by a patient's overall care goals, prognosis, and preferences, rather than a one-size-fits-all approach. One unique aspect of this study is its focus on a Middle Eastern palliative care setting, where cultural, institutional, and clinical practices may differ from Western settings. Future research should focus on identifying specific clinical scenarios in which antibiotics provide meaningful symptomatic relief, developing standardized guidelines for antibiotic initiation in palliative settings, and evaluating the long-term impact of antibiotic prescribing on patient-centered outcomes. Until then, a more judicious approach to antibiotic use, emphasizing individualized care goals and symptom-focused management, may help optimize EoL care while avoiding unnecessary medicalization.

### Limitations

This study has several limitations that must be acknowledged: The study's retrospective nature restricts causal inferences between antibiotic use and symptom improvement. Documentation variability in medical records may have influenced the accuracy of symptom assessment. Conducted in a single cancer center in Saudi Arabia, the findings may not be generalizable to other settings, particularly those with different healthcare systems or cultural practices. Symptomatic improvement was assessed based on clinician documentation rather than standardized patient-reported outcomes, introducing potential bias. The study focused on symptomatic improvement at day 3, which may not reflect the longer-term impact of antibiotic therapy or the natural progression of symptoms. A potential limitation of this study is the subjective nature of symptom assessment in retrospectively reviewed medical records. Clinician interpretation of symptoms and infection-related distress may have varied, leading to inconsistencies in documentation.

Additionally, the absence of standardized protocols for infection assessment in palliative care may have influenced treatment decisions and outcome evaluations. Due to the study's retrospective nature, variations in clinician documentation and diagnostic criteria may have influenced the recorded symptomatology. The low involvement of infectious disease specialists may have influenced prescribing patterns and outcomes, highlighting a need for greater interdisciplinary collaboration in palliative care. The high reliance on broad-spectrum agents like piperacillin/tazobactam may not represent all antibiotic practices globally, and their use may carry higher risks of adverse effects and antimicrobial resistance. Despite these limitations, this study provides valuable insights into the patterns and implications of antibiotic use in palliative care, particularly in a Middle Eastern context, and highlights areas for improvement in clinical practice and research.

## Conclusion

This study adds to the growing body of evidence on antibiotic use in EoL care, highlighting its widespread use but limited symptomatic benefit in advanced cancer patients. While antibiotics could relieve certain symptoms, their overall utility in this population remains unclear. The findings underscore the importance of a multidisciplinary approach to antibiotic decision-making, ensuring that the potential benefits align with the patient's care goals and minimize harm. Efforts should be made to develop evidence-based guidelines tailored to palliative care settings, particularly in underrepresented regions like the Middle East. Future research should focus on prospective studies evaluating standardized criteria for antibiotic initiation, the role of interdisciplinary teams in prescribing decisions, and the long-term impact of antibiotics on patient-centered outcomes such as quality of life.
